# Assessment of Under Nutrition of Bangladeshi Adults Using Anthropometry: Can Body Mass Index Be Replaced by Mid-Upper-Arm-Circumference?

**DOI:** 10.1371/journal.pone.0121456

**Published:** 2015-04-14

**Authors:** Tania Sultana, Md. Nazmul Karim, Tahmeed Ahmed, Md. Iqbal Hossain

**Affiliations:** 1 Center for Nutrition and Food Security, International Centre for Diarrheal Disease Research, Bangladesh (icddr,b), Mohakhali, Dhaka 1212, Bangladesh; 2 Department of Epidemiology and Preventive Medicine; Faculty of Medicine Nursing and Health Science, Monash University, The Alfred, 99 commercial road, Melbourne 3004, Australia; 3 Dhaka Hospital, icddr,b, Mohakhali, Dhaka 1212, Bangladesh; Vanderbilt University, UNITED STATES

## Abstract

**Background and Objective:**

Body-mass-index (BMI) is widely accepted as an indicator of nutritional status in adults. Mid-upper-arm-circumference (MUAC) is another anthropometric-measure used primarily among children. The present study attempted to evaluate the use of MUAC as a simpler alternative to BMI cut-off <18.5 to detect adult undernutrition, and thus to suggest a suitable cut-off value.

**Methods:**

A cross-sectional study in 650 adult attendants of the patients of Dhaka-Hospital, of the International Centre for Diarrheal Disease Research, Bangladesh (icddr,b) was conducted during 2012. Height, weight and MUAC of 260 male and 390 female aged 19–60 years were measured. Curve estimation was done to assess the linearity and correlation of BMI and MUAC. Sensitivity and specificity of MUAC against BMI<18.5 was determined. Separate Receiver-operating-characteristic (ROC) analyses were performed for male and female. Area under ROC curve and Youden's index were generated to aid selection of the most suitable cut-off value of MUAC for undernutrition. A value with highest Youden's index was chosen for cut-off.

**Results:**

Our data shows strong significant positive correlation (linear) between MUAC and BMI, for males r = 0.81, (p<0.001) and for females r = 0.828, (p<0.001). MUAC cut-off <25.1 cm in males (AUC 0.930) and <23.9 cm in females (AUC 0.930) were chosen separately based on highest corresponding Youden's index. These values best correspond with BMI cut-off for under nutrition (BMI <18.5) in either gender.

**Conclusion:**

MUAC correlates closely with BMI. For the simplicity and easy to remember MUAC <25 cm for male and <24 cm for female may be considered as a simpler alternative to BMI cut-off <18.5 to detect adult undernutrition.

## Introduction

Nutrition related issues are often neglected in adults living in low-income countries. A suitable indicator of nutritional status in adults is needed. Body mass index (BMI) has been widely used as an indicator of nutritional status in adults.

BMI is an objective indicator of generalized adiposity [1] and is the most widely used [2–6] anthropometric indicator for assessing nutritional status of adults. It is a non-invasive technique used for nutritional surveys [3, 5], and can also provide insight into the socioeconomic status of a population, particularly in developing countries [7–9]. BMI <18.5 kg/m^2^ is considered an indicator of undernutrition and it predicts an individual's morbidity or other physiological and functional impairments [10]. However, BMI has some drawbacks and practical limitations as a measurement tool in the quick assessment of individuals (e.g. debilitated, disabled or acutely ill patients). It is not always possible to measure weight or height, particularly in debilitated and immobile patients. The reason is nearly always that patients cannot be taken out of their beds to be weighed and/or cannot stand for height measurements. BMI is particularly inappropriate for pregnant women. Due to the extra weight of the fetus, other products of conception, and added maternal tissue. Furthermore, in resource limited health settings and population-based surveys, accurate measurements of height and weight require reasonably large logistical mobilization.

In such situations, a reasonable alternative would be mid-upper arm circumference (MUAC), the most common anthropometric measure used to evaluate the nutritional status of children [11–14]. In children MUAC has been shown to be very useful in the assessment of nutritional status in community settings [15, 16]. It is a simpler measure than BMI, requiring minimum equipment and has been demonstrated to predict morbidity and mortality in 6–59 months old Senegalese children [17]. Such measurements have recently been used in the diagnosis of adult malnutrition in hospitals [18] and it is found that low MUAC better predicts mortality than low BMI in Dutch older adults [19]. Vlaming et al [20] reported that MUAC has the advantage of being easier to measure than BMI or any other height-weight derived indices. A reasonably close relationship between MUAC and BMI has been demonstrated in normal adult populations from a number of developing countries. Its simplicity and ease of use make it a candidate for use in adult nutritional assessment. The Present study attempted to evaluate the use of MUAC as a reliable alternative to BMI to detect undernutrition, and thus to suggest a suitable cut-off value for the identification of adult undernutrition.

## Methods

### The setting, subjects and data collection

It was a cross-sectional study and data were collected prospectively from 650 attendants of patients of the Dhaka Hospital of the International Centre for Diarrheal Disease Research, Bangladesh (icddr,b) during 2012. The study participants were 260 male and 390 female adults aged between 19 to 60 years. No socio-economic parameter was considered in their selection. Athletes, persons with a mental illness or disability, hormonal or any apparent congenital dysmorphism, and pregnant women were not enrolled in this study. Based on selection criteria, the first 5 to 6 consecutive elegible attendents (from 9 am to 4 pm during Sunday to Thurstday excluding the holidays) were approached; those who consented were included in the study. Age, educational status, and occupation were inquired through a questionnaire. Weight (to nearest 10 g), standing height (to nearest 100 g), MUAC (to nearest 1mm) in sitting or standing posture were measured following standard procedures using calibrated instrument. During weight taking each subject was asked to stand without any heavy clothing, relaxed with arms at the sides, feet positioned close together and weight evenly distributed across feet. Height was measured by a stadiometer without shoe and sock, while the feet were placed together with heels; back of the heels, buttocks and shoulder blades touched the back plate/stick and head was positioned in the Frankfurt horizontal plane. MUAC was measured with a non-stretchable MUAC measuring tape at a point equidistant between the acromion process of the left scapula and the olecranon process of the left ulna. All anthropometric measurements were taken twice and the average was recorded. If the measurements varied by more than 100 g for weight, 0.5 cm for height and 0.2 cm for MUAC a third measurement was made. The average of the nearest two measures was recorded. BMI was calculated using the standard formula: weight in kg/(height in m)^2^. The BMI cut-off point of <18.5 was used to identify adult undernutrition. Nutritional status was evaluated using both BMI and MUAC.

### Ethical Considerations

Aproval from the Institutional Review Board (IRB) of the State University of Bangladesh and permission from the Medical Director of the Dhaka Hospital of icddr,b were granted. Informed verbal consent (with IRB approval) from the participants was taken prior to data collection. Confidentiality was assured and maintained. The verbal consent process involved an education and information exchange (explanation of the research process and what the participant would do if s/he agrees to participate in the study) took place between the research assistant and the potential participant in the presence of a witness. The research assistant specifically explained/discussed the anthropometry measurement procedures, a non-invasive procedure. Verbal consent was documented by recording a check mark in the questionnaire which was again shown to the participants.

### Statistical Analysis

Data were analyzed using IBM SPSS statistics version 22 and MedCalc 6.1. Descriptive statistics were generated for demographic variables, all measurements (weight, height and MUAC), and BMI. Curve estimation was done to assess the linearity of BMI and MUAC. Subsequently correlation analysis between BMI and MUAC was performed. Receiver operating characteristics (ROC) analysis was done and ROC curve of (MUAC) based on BMI <18.5 was generated separately for male and female respondents. Coordinates point for each MUAC value was tabulated. Area under ROC curve (AUC) with and its 95% CI were generated separately for male and female. Optimal cutoff point was found by maximizing the sum of sensitivity and specificity. A MUAC value with highest Youden's index was chosen for cutoff.

## Results

Of the 650 recruited individuals, 260 were male and 390 were female. The mean ± standard deviation (SD) age of the male and female participants was 31±8.1 and 25±5.9 years respectively, and a monthly family income of 8879±5778 taka. Table 1 describes the sex specific mean±SD value of different anthropometric variables.

**Table 1:** Anthropometric characteristics by sex.

Characteristics	Male, n = 260 Mean ± SD	Female, n = 390 Mean ± SD	t	p value
**Age (years)**	31.1 ± 8.1	25.4 ± 5.9	10.234	< 0.001
**Weight (kg)**	52.85 ± 8.95	47.27 ± 9.91	7.302	< 0.001
**Height in (cm)**	160.6 ± 6.2	148.8 ± 5.8	24.904	< 0.001
**Body mass index (kg/m** ^**2**^ **)**	20.49 ± 3.29	21.27 ± 3.89	-2.663	< 0.008
**MUAC (cm)**	25.9 ± 2.9	25.3 ± 3.3	2.265	< 0.024

**Fig 1:**
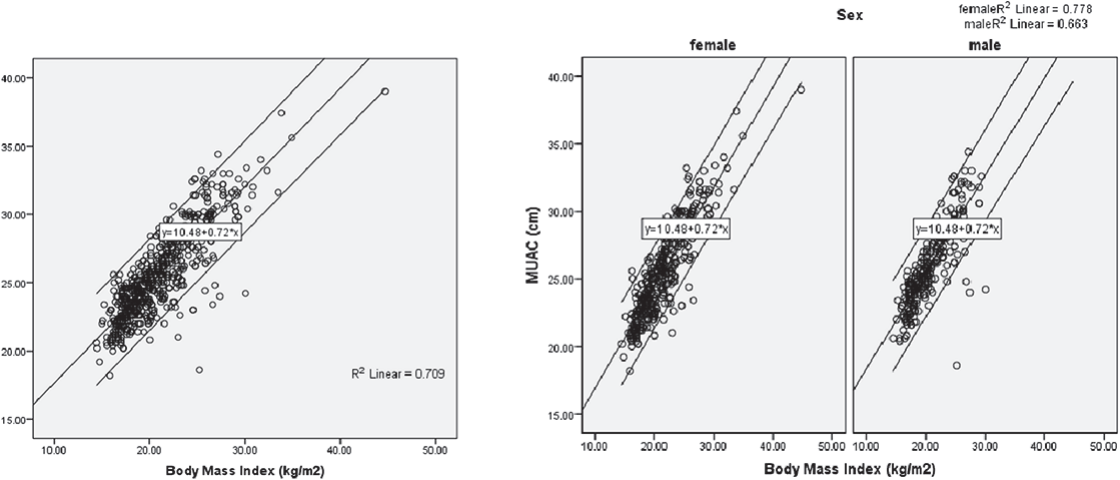
Curve estimation for assessing linear relationship between MUAC and BMI.

**Fig 2:**
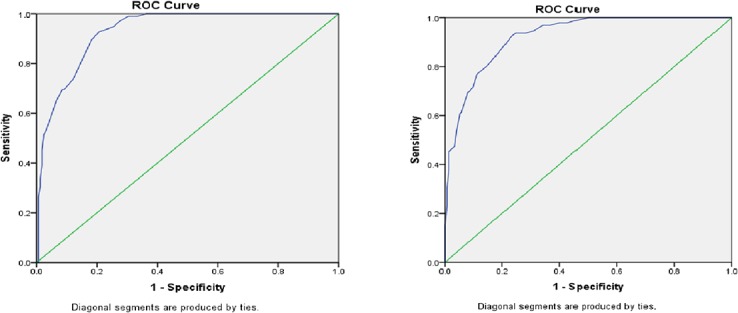
Receiver operating curve of mid-upper-arm-circumference (MUAC) based on body mass index (BMI <18.5) for male (left panel) female (right panel).

Using the Curve Estimation procedure, comparison between linear and quadratic models for the relationship between MUAC and BMI were performed. Linear model was found to be adequate because its coefficients (B 0.743, p <0.001) and residuals were highly significant independent of the fit values. The quadratic model did not have similar significant coefficient and fit values. Hence linear correlation was used for assessing relation between the two measurements. Fig 1 illustrates the linear relationship between MUAC and BMI. (MUAC = 10.481 + 0.718* BMI). Table 2 demonstrates highly significant positive correlation between MUAC and BMI among both males (r 0.814 p < 0.001) and females (r 0.882 p < 0.001). To locate the optimal cut-off values of MUAC the ROC analysis was performed and ROC curve was generated separately for men and women. Based on BMI < 18.5 the area under the receiver operating curve (AUC) of MUAC for male (0.93; 95% CI 0.90–0.96) and female (0.92; 95% CI 0.90–0.95) was found high (Table 3 and Fig 2). Coordinate points for each MUAC value was generated. Cut-off point was found by maximizing the sum of sensitivity and specificity. MUAC value of 25.1 cm (Youden's index 0.72) for male and 23.9 cm (Youden's index 0.69) for female were found to be the suitable cut-off points for detecting malnutrition as they posses' highest Youden's index.

**Table 2:** Correlations of mid-upper-arm-circumference (MUAC) with body mass index by sex.

	Pearson correlation(two tailed)	p value
**Male**	0.814	<0.001
**Female**	0.882	<0.001

**Table 3:** Area under the receiver operating curve (AUC) for mid-upper-arm-circumference (MUAC) based on body mass index < 18.5 for male and female separately.

Variable	Area under AUC Mean (Standard error)	95% Confidence interval of AUC	p value
**Male**	0.930 (0.015)	0.901–0.959	0.001
**Female**	0.923 (0.014)	0.897–0.950	0.001

The MUAC cut-off for male (25.1 cm) shows a sensitivity of 92.6% and specificity of 79.6% and the cut-off for female (23.9 cm) shows a sensitivity and specificity of 92.6% and 76.46% respectively (Table 4).

**Table 4:** Evaluation of screening test of nutritional status (BMI < 18.5) by mid-upper-arm-circumference (MUAC) based on highest Youden’s index.

Gender	Youden's Index	Cut-off^1^	Sensitivity	1—Specificity	LPR^2^	LNR^3^
**Male**	.720	25.1	.926	.206	4.495	.093
**Female**	.692	23.9	.926	.234	3.960	.096

^1^Positive if less than or equal to

^2^ LRP: Likelihood Ratio for positive test

^3^LNR: Likelihood Ratio for negative test

## Discussion

Our results show a strong correlation between BMI and MUAC. This finding lays the ground for the suitability of MUAC as an indicator of nutritional status in adult. ROC analyses also echo this finding. Although both BMI and MUAC could be used to evaluate nutritional status, MUAC may be preferred for its simplicity. Measurements of arm circumference have long been known to reflect changes in body weight [21]. MUAC requires neither mathematical derivation nor expensive equipment. The measurement is easy to perform [22, 23] even on the most debilitated person. MUAC emerges as a useful measure of nutritional status due in part to its applicability in nearly all acutely ill patients whom measurements of weight and height may be inappropriate or impossible [24]. It is being increasingly recognized as an effective measure of screening for poor nutritional status in adults [25], and shown as a valuable alternative to BMI in identification of chronic energy deficiency in adult male non-tribal slum dwellers in West Bengal, India [26]. Further, it was found as a better predictor of poor health status and morbidity among Bengali adults in Kolkata, India [26], although the assessment of the relationships between anthropometry and morbidity in the present study is beyond the remit.

MUAC is particularly suitable for large scale studies and surveys, as it can be measured with limited resources for human population surveys, especially among rural populations of developing countries. It can be used as a substitute alternative to BMI, because a MUAC cut-off of 23 cm in male and 22 cm in female was found useful in determining malnutrition among adults in developing countries [3]. It has been demonstrated to be an efficient screening technique for the assessment of nutritional status in a variety of ethnic groups [27, 28]. In Sudanese population, MUAC was found easier to perform than and correlated very well with BMI for screening of undernourished adults [29].

In the present study, receiver operating characteristics analysis determined 25.1 cm as the best cut-off for male undernutrition and 23.9 cm for female. Analyses of anthropometric data from nine adult surveys conducted in Asia, Africa and the Pacific calculated a series of MUAC cut-off points to allow for the screening of individual adults under extreme famine conditions [28]. They proposed that a MUAC <20.0 cm for men and <19.0 cm for women represents grade 4 malnutrition. Extreme wasting is said to correspond to MUAC values less than 17.0 cm and 16.0 cm for men and women respectively. However, our cut-off only differentiates undernutrition from normal nutrition. Chakraborty et al. reported a MUAC of 24.0 cm as the cut-off for detecting chronic energy deficiency for male Kolkata slum dwellers [30].

As the measurement of MUAC requires minimal equipment and is easy to perform even on the most debilitated individuals it is appropriate for nutritional status screening during famine and emergencies. Major determinants of MUAC, arm muscle and subcutaneous fat, are both important determinants of survival in starvation [31]. MUAC is less affected than BMI by the localized accumulation of excess fluid (edema, ascites) common occurrences during famines and similar situations [21].The above also justify the use of MUAC as a substitute anthropometric indicator or a compliment index to the BMI. One important consideration is the ethnic variation of physical configuration and built. There is strong evidence that, body fat distribution varies across ethnic entities [1]. The relationship between overall adiposity (e.g. measured by BMI) and regional adiposity, measured as body circumferences (waist, MUAC) and skin fold thickness, was also shown to vary according to the population [32]. Although BMI value <18.5 is indicative of undernutrition across ethnic groups [11], studies have clearly shown significant ethnic differences in regional adiposity and body composition measures (e.g. % body fat) at the same level of BMI [33]. Hence, ethno-specific cut-offs for MUAC to detect undernutrition is warranted as well. However, the recommended MUAC cut-off value of <25.1 cm and 23.5 (for male and female respectively) to define undernutrition in population may not be the most appropriate for all ethnic groups. A cut-off point of 24.0 cm was reported to be suitable in a recent study from the south of India [34]. Similarly, another recent study of non-tribal adult slum dwellers of Bengali ethnicity in West Bengal, India, reported a MUAC value of 24.0 cm to be the most appropriate cut-off point for identifying adult undernutrition. The cut-off derived from our data was tested with ROC analysis to assess its predictive capacity for both men and women. This proposed cut-off point is likely to have large public health implications, especially with respect to primary healthcare dealing with situations where malnutrition is prevalent.

The assessment of MUAC requires no equipment apart from a tape measure. As the index is the actual measurement itself, mathematical manipulation of the measurement obtained is not necessary. Despite the convenience and superiority over height-weight dependent indices, the observer should be aware of artifacts, which can result in an erroneous estimate and some degree of intra-observer variability. Careful training and supervision should be maintained in order to prevent wrapping the measuring tape too tightly or too loosely etc.

One obvious limitation of the cut-off proposed in the present study is that the data came from one source location which may not comprehensively represent the entire population. Thus, validation studies are needed with a larger and more representative sample that includes participants’ medical history.

We conclude that MUAC correlates closely with BMI and appears to accurately detect adult undernutrition as defined by BMI. For the simplicity and easy to remember, MUAC <25 cm for male and <24 cm for female may be considered as a simpler alternative to BMI cut-off <18.5 to detect adult undernutrition.
